# A role for cutaneous gamma delta T cells in the development of systemic lupus erythematosus

**DOI:** 10.1186/ar4639

**Published:** 2014-09-18

**Authors:** Mehran Ghoreishi, Jan P Dutz

**Affiliations:** 1 University of British Columbia, Vancouver, BC, Canada

## Background

Ultraviolet (UV) light exposure promotes the development of cutaneous lupus and can induce flares of systemic lupus erythematosus. Similarly, UV light can induce lupus in genetically prone strains of mice. Nonobese diabetic (NOD) mice prone to autoimmune disease demonstrate lupus-like disease after repeated combined UV irradiation and topical application of TLR-7 agonist imiquimod (IMQ). In contrast, repeated topical application of a Toll-like receptor (TLR) 7/8 agonist promotes psoriasiform skin lesions in nonautoimmune mice such as Balb/c. The cellular and molecular determinants of these processes are still unknown.

In this study we have compared the skin lesions induced by repeated topical IMQ in Balb/C *VS *NOD mice. Gamma delta T cells are innate immune cells that bridge innate and adaptive immunity in part by modulating dendritic cell maturation. We have thus explored the role of gamma delta T cells in the induction of psoriasis and SLE following topical TLR 7/8 agonist application.

## Methods

NOD and Balb/C mice received 70 mg topical IMQ 5% cream on shaved back skin daily for 5 days. Skin, draining lymph nodes (LNs) and sera were obtained for histological and serological analysis.

## Results

Balb/c mice but not NOD mice developed thickened erythematous and scaly skin lesions similar to psoriasis. Histological examination revealed acanthosis, parakeratosis and papillomatosis resembling psoriasis in Balb/c mice but not in NOD mice. IL-17-producing T cells were increased in the dermis and skin draining LNs of Balb/C mice after 5 days topical IMQ, whereas the frequency of IFNγ-producing gamma delta T cells was higher in NOD mice. Serum collected 24 hours after treatments with IMQ showed elevation of Th1 cytokines in NOD mice, but not in Balb/C mice. Repeated IMQ increased the relative frequencies of granulocytes in the skin and skin draining LNs in Balb/c but not in NOD mice. In contrast, the frequency of macrophages was increased in NOD skin.

## Conclusions

Our work suggests that a predominant Th1 response, orchestrated in part by gamma delta T cells in response to TLR7/8 agonism in the skin, may predispose to SLE-like autoimmunity.

